# The inevitable colonisation of Singapore by Zika virus

**DOI:** 10.1186/s12916-016-0737-9

**Published:** 2016-11-21

**Authors:** Dale Fisher, Jeffery Cutter

**Affiliations:** 1 Division of Infectious Diseases, National University Hospital, Singapore, Singapore; 2 Yong Loo Lin School of Medicine, National University of Singapore, Singapore, Singapore; 3 Communicable Diseases Division, Ministry of Health, Singapore, Singapore

**Keywords:** Zika, ZIKV, Singapore, Asia, Epidemiology, Outbreak

## Abstract

Singapore is endemic for Dengue virus, with approximately 10,000 to 20,000 annual cases reported in recent years. In 2012, Chikungunya was introduced, although the numbers of cases reported is much fewer. The current Zika virus pandemic originating in Brazil represents a threat to all regions with Aedes mosquitoes, particularly those well connected by travellers. In this respect, it was felt inevitable that Singapore would eventually realise its third endemic flavivirus. In late August 2016, a primary care practitioner observed a cluster of geographically linked patients attending with fever and rash. This resulted in the first identification of locally transmitted Zika in Singapore on August 27, 2016. This prompted a robust response in an attempt to stop further spread, which continued for approximately 10 days until a large number of laboratory-confirmed cases were found as a result of active case finding. Surprisingly, the strain was later identified to be of Asian lineage and distinct from that originating in the Americas, prompting speculation over the epidemiology of this under recognised virus in Asia.

## Background

By early October 2016, 73 countries had reported autochthonous transmission of Zika virus (ZIKV) since 2007 [1]. Singapore joined this list on August 27, 2016, and while this first identified case signalled a large outbreak, the subsequent realisation that this outbreak was unrelated to that of the Americas raises many questions regarding ZIKV epidemiology in Southeast Asia.

## Tracking the epidemiology in countries

Internationally, the identification of ZIKV cases is heavily influenced by the sophistication of health systems, affordable, convenient availability of testing, the health seeking behaviour of individuals, and the severity of the illness, together with treatment options. Given that this is generally a mild (usually asymptomatic) disease with no treatment and which occurs in tropical regions, when dealing with the inevitable underreporting of ZIKV, international organisations categorise affected countries to describe current reports without dismissing past reported cases. The European Centre for Disease Prevention and Control, the World Health Organization, and the US Centres for Disease Control and Prevention have categories to describe countries with recent or current outbreaks, sporadic or endemic disease, or past transmission, and countries at risk including risk stratification based on the presence of Aedes mosquitos or Dengue virus (DENV) [1–3]. However, these strategies largely manage the varied efforts involved in case finding and testing (both between countries and temporally within countries). Vector control, personal protective efforts and, to some extent, herd immunity can end an outbreak but will likely lead to a baseline low level endemicity. Elimination is possible in areas of low population density as has been the experience with DENV in Australia. However, where elimination is achieved, vulnerability remains and reintroduction is the norm [4].

Of the 73 countries hosting ZIKV transmission in the last decade, 64 have documented cases in the last 9 months and 58 in the last 3 months. It is highly likely, however, that countries absent from more recent lists have accepted ongoing transmission but have higher health priorities precluding substantive ZIKV surveillance. Countries with Aedes mosquitoes generally have endemic DENV and, in practice, neither can be sustainably eradicated [4, 5].

The risk of ZIKV introduction to Singapore and the likelihood of it becoming endemic has been well recognized locally for some years. Dengue is endemic in Singapore, with disease predominantly in adults. Recent outbreaks have resulted in over 10,000 notified cases per year, including a peak of 22,170 cases in 2013. This is despite highly successful vector control programmes legislated since 1966 [6]. This paradoxical outcome is believed to be due to decreased childhood exposure and therefore a lack of immunity in adulthood when exposure is more likely to have a symptomatic presentation.

Combining this setting, apt for a new mosquito-borne flavivirus, with Singapore’s global connectedness of approximately 15 million tourists per year, the issue was ‘when’, rather than ‘if’, Singapore would join the list of ZIKV affected countries [7]. However, the expectation was that it would be the strain originating in the Americas, which is central to the current international spread.

## ZIKV surveillance in Singapore

In an effort to identify the apparently inevitable introduction, Singapore had undertaken regular routine surveillance for ZIKV since 2014 after the outbreak in French Polynesia in 2013 [8]. Samples negative for DENV diagnostic testing were used for ZIKV surveillance. This surveillance was scaled up significantly after reports in January 2016 describing the large ZIKV outbreak in Brazil and its possible association with microcephaly.

Ministry of Health advisories alerted doctors to test travellers from ZIKV-affected areas with compatible symptoms. All blood samples were tested at the National Public Health Laboratory using RT-PCR. Furthermore, public travel advisories gave information about avoiding ZIKV infection and also presenting early if symptoms were present on return.

## Managing the first cases

In May 2016, a Singapore resident returning from Brazil became the first (imported) confirmed case prompting containment efforts. Vector control operations around his residence were intensified for a week. Doctors were asked to refer patients with compatible symptoms and an epidemiological link to the area. The man was isolated in hospital until he was no longer viraemic and no further cases were detected.

The first case of local transmission of ZIKV was reported on August 27, 2016, and this time prompted enhanced containment efforts comprising screening of those living or working around confirmed cases, referral of suspect cases to hospitals for ZIKV testing, isolation of viraemic cases, and intensive vector control. Over 200 positive diagnoses were made in the first week, including over 50 diagnoses from retrospective testing of patients with recent symptoms. After 2 weeks, over 300 cases had been identified. Clearly, the ‘first case’ was not the index: other clusters geographically separate in Singapore were found and containment involving isolation of cases was discontinued after 10 days. Intensive vector control around clusters of cases continued, including community engagement to prevent mosquito breeding.

Going forward, clinical management involving ZIKV has been prioritized to symptomatic pregnant women, with testing fully subsidized. A national expert advisory group has provided guidelines for the clinical management of ZIKV infection in pregnancy. Surveillance has been enhanced via selected primary care clinics and tests on DENV-negative serum will continue as previously.

## Why an outbreak of the Asian lineage now?

It is not clear why this outbreak occurred. The case in May (a traveller from Brazil) carried the strain that is part of the world pandemic. However, the August outbreak strain is a distinctly different Asian lineage circulating since the 1960s [9].

It is quite possible that ZIKV has been circulating for decades in Asia, and even in Singapore, but the absence of specific tests for antibodies denies the possibility of a seroprevalence study. In fact, the major issue in defining such a test (or therapeutic) is cross reactivity with other flaviruses, particularly DENV [10]. Indeed, some of the cases over the years diagnosed serologically as a DENV infection could have been ZIKV.

Whether or not there is a baseline low level of ZIKV in Singapore does not replace the fact that this was an outbreak starting at least some generations prior to August 27; this may have been a chance occurrence or it may be a result of ongoing urbanization across Asia, with adaptation by Aedes species of mosquito and possibly evolution of the ZIKV genotype, behaving similarly, both virologically and epidemiologically, to Chikungunya and DENV [11].

## Conclusion

In Singapore, more than 1 month after the reporting of the first confirmed locally acquired case and after the laboratory confirmation of over 400 cases, case numbers have dropped to below 10 per week. The public and the medical community have accepted the inevitability of the disease, but because of its mild (usually asymptomatic) nature, understanding what happened and tracking the epidemiology of ZIKV will be a challenge. This would be acceptable if not for the potential impact on the unborn and occasional neurological sequelae in adults. ZIKV will likely persist in Singapore and cause occasional outbreaks and new strains will periodically be introduced. Understanding the molecular epidemiology and the clinical impact, establishing specific antibody tests and multiplex PCRs, and striving for treatments, particularly vaccines, all form part of the international research agenda [12]. The current response capacity to counter ZIKV is limited to vector control. The targeted knowledge and tools once available will be a welcome addition to health system managers and clinicians in the future.
